# Not All IgA Nephropathy are the Same: Clinical Implications of Recurrent Versus *de novo* Disease Posttransplant

**DOI:** 10.1016/j.ekir.2025.06.001

**Published:** 2025-06-07

**Authors:** Mythri Shankar, Nada Alachkar

**Affiliations:** 1 Department of Nephrology, Institute of Nephro-Urology, Bengaluru, Karnataka, India; 2 Division of Nephrology, Department of Medicine, The Johns Hopkins University School of Medicine, Baltimore, Maryland, USA

See Clinical Research on Page 2659

IgA nephropathy (IgAN) is a leading cause of primary glomerulonephritis worldwide, with up to 40% of patients progressing to end-stage kidney disease within 20 years.^1^ Kidney transplantation remains the best treatment for end-stage kidney disease, improving both survival and quality of life. However, long-term graft survival has plateaued, with recurrent glomerulonephritis, especially IgAN, contributing significantly to late allograft failure, along with chronic rejection and patient death.^2^

Early registry data, including from the Australia and New Zealand Dialysis and Transplant registry^3^ and the US Renal Data Sysytem^4^ suggested a low incidence of graft loss due to IgAN recurrence. However, these figures may underestimate the burden because of underdiagnosis and misinterpretation of its subtle, often asymptomatic presentation, commonly limited to microscopic hematuria, resulting in delayed recognition.^5^

A more recent large-scale, long-term study^1^ has challenged earlier views, showing that recurrent IgAN markedly increases the risk of graft failure, particularly beyond 10 to 15 years posttransplant. Updated Australia and New Zealand Dialysis and Transplant registry^2^ analyses revealed a 2-fold higher risk of graft failure in patients with recurrent IgAN. Similarly, the multinational TANGO project found recurrence rates as high as 23% at 15 years, with a significantly increased hazard ratio for graft loss.^6^

Furthermore, the Banff GN working group investigated the impact of recurrent IgAN on the allograft survival and analyzed the predictive value of the MEST-C score in posttransplant IgAN. The group reported worse outcomes in patients with recurrent IgAN. IgAN recurrence strongly increased the risk of death-censored graft failure (adjusted hazard ratio: 5.10 [95% confidence interval: 2.26–11.51]; *P* < 0.001). In addition, they found that higher MEST-C score was associated with death-censored graft failure (adjusted hazard ratio: 8.57 [95% confidence interval: 1.23–59.85; *P* = 0.03] and 61.32 [95% confidence interval: 4.82–779.89; *P* = 0.002] for score 2–3 and 4–5 versus 0, respectively); and so were the single components endocapillary hypercellularity, interstitial fibrosis/tubular atrophy, and crescents (*P* < 0.05 each).^7^

Protocol biopsies from centers such as the Mayo Clinic have revealed even higher rates of histological recurrence, emphasizing that clinical diagnosis alone may miss early or subclinical recurrence. In these studies, histological recurrence occurred in > 50% of recipients within 5 years, and was strongly associated with subsequent graft failure.^8^

The recent study by Haas *et al.*,^9^ offers valuable insights into the differences between recurrent and *de novo* IgAN in kidney transplants and highlights key pathological and clinical predictors of graft outcomes. This retrospective single-center study reviewed 264 transplant biopsies over a 14-year period, focusing on patients with glomerular IgA deposits and distinguishing recurrent IgAN (*n* = 127) from *de novo* IgAN (*n* = 46). The investigators further characterized these 2 groups using Oxford MEST-C scoring, intensity of C3 staining, and clinical data at biopsy. Follow-up data allowed evaluation of graft outcomes in 74 patients.^9^

The importance of this study lies in its attempt to tease apart the clinical and pathological nuances between recurrent and *de novo* IgAN, entities that are often lumped together in the literature. Recognizing these distinctions is critical in clinical practice for prognostication and individualized management.

The study demonstrates that recurrent IgAN is more likely to present with active histological lesions—mesangial hypercellularity (M1), endocapillary hypercellularity (E1), crescents (C1/2), and > 1+ glomerular C3 deposition—when compared with *de novo* disease. This suggests a more inflammatory phenotype, aligning with what is often observed in native kidney IgAN. Interestingly, the degree of chronic damage (S and T scores) was similar between the groups, likely reflecting the similar posttransplant time at which biopsies were performed.

Crucially, recurrent IgAN was associated with worse graft outcomes, although statistical significance was narrowly missed (*P* = 0.106). When examining predictors of graft loss, multivariable analysis confirmed that higher serum creatinine, greater proteinuria, T1/2 and C1/2 lesions, and C3 deposition > 1+ were independently associated with poorer outcomes.

These findings support the premise that recurrent IgAN is not merely a histologic curiosity but a clinically meaningful process that can jeopardize graft survival. Notably, the Oxford T and C scores maintained prognostic significance even after adjusting for serum creatinine and proteinuria, thereby reinforcing the relevance of histopathology in prognostication.

Compared with recurrent cases, *de novo* IgAN appeared less aggressive. Fewer biopsies showed M1, E1, or C1/2 lesions, and most had minimal C3 deposition. Clinically, patients with *de novo* IgAN also tended to have lower proteinuria and serum creatinine at biopsy. Although follow-up numbers were limited, graft survival was generally better in the *de novo* group (Table 1).

**Table 1:** Comparison of recurrent versus de novo IgA nephropathy in kidney transplants

Feature	Recurrent IgAN	*De novo* IgAN
Number of biopsies (*n*)	127	46
Previous native kidney diagnosis of IgAN	Present	Absent
Time posttransplant (mean number of months)	∼91	∼93
Mesangial hypercellularity (M1)	45%	17%
Endocapillary hypercellularity (E1)	28%	4%
Segmental glomerulosclerosis (S1)	56%	46%
Tubular atrophy / interstitial fibrosis (T1–2)	51%	41%
Presence of crescents (C1–2)	16%	2%
Glomerular C3 staining > 1+	59%	15%
Concurrent cell-mediated Rejection	19%	35%
Biopsies done for proteinuria	28%	13%
Graft loss (among those with follow-up)	46%	25%
Proteinuria at biopsy (U P/C > 1)	84%	40%
Interpretation	Typically, more inflammatory, aggressive, and associated with worse outcomes	Milder histology, often with better prognosis

IgAN, IgA nephropathy; U P/C, urinary protein-to-creatinine ratio.

This supports the idea that *de novo* IgAN may often be incidental or less pathogenic, possibly stemming from mechanisms distinct from recurrent IgAN. Nonetheless, close monitoring is essential, because some cases may still progress, especially with inflammation and proteinuria.

A novel aspect of this study is its evaluation of C3 deposition as a surrogate marker of complement activation in the graft. In line with native IgAN studies, C3 > 1+ staining correlated with more active lesions and worse graft outcomes. Although not all associations remained statistically significant in multivariable analysis, the hazard ratios suggest clinically meaningful risk. This may have therapeutic implications, particularly because complement-targeting agents such as iptacopan are being tested in native IgAN. This highlights the potential role of C3 immunofluorescence as an adjunct marker to stratify disease severity and prognosis in the transplant setting, adding nuance to traditional MEST-C scoring.

In routine nephrology practice, this study highlights several key considerations as follows:

1. Native disease documentation: Differentiating recurrent from *de novo* IgAN has implications for prognosis and treatment. Accurate native disease records are crucial, especially because 27% of cases in this study lacked them.
2. Routine immunofluorescence and MEST-C scoring: Allograft biopsies with IgA deposits should be assessed with standardized MEST-C scoring and complement deposition, consistent with the Kidney Disease: Improving Global Outcomes’ risk prediction model.
3. Risk stratification and follow-up: Patients with M1, E1, C1/2 lesions or C3 > 1+ deposition need close surveillance and potentially earlier intervention. Proteinuria monitoring remains vital, given its strong association with graft outcomes.
4. Revisiting donor selection: An unexpected finding was that living-related donor transplants had worse outcomes in recurrent IgAN compared with deceased donors, even after adjusting for creatinine and proteinuria. Although not definitive, this prompts reconsideration of living-related donor in patients with IgAN with high-risk features or family history—pending further validation.

Despite its strengths, the study has limitations inherent to its retrospective, single-center design. The modest sample size for outcome analysis, especially in the *de novo* group, limits generalizability. Moreover, protocol biopsies were not performed, precluding estimation of true recurrence rates and detection of subclinical disease. A multicenter prospective study with protocol biopsies and standardized clinical-pathologic correlation would help address these gaps.

Further, understanding the molecular signatures and immune profiles of recurrent versus *de novo* IgAN may yield insights into divergent pathogenesis and identify novel therapeutic targets. The potential role of anticomplement therapies in posttransplant IgAN also deserves exploration.

This study meaningfully contributes to understanding of IgAN in kidney transplant recipients, emphasizing the importance of distinguishing recurrent from *de novo* disease. It reinforces the prognostic utility of the Oxford MEST-C scoring system and glomerular C3 staining in the transplant setting. For clinicians, the take-home message is clear: recurrent IgAN is not benign. It necessitates vigilant follow-up, robust risk stratification, and possibly targeted therapeutic approaches. As the treatment landscape for IgAN evolves, transplant nephrologists must integrate these findings into personalized posttransplant care to optimize long-term graft survival.

## Disclosure

All the authors declared no competing interests.
